# The Significance and Mechanism of Cerebral Enlarged Perivascular Space in Amyotrophic Lateral Sclerosis

**DOI:** 10.3390/ijms26199474

**Published:** 2025-09-27

**Authors:** Bo-Ching Lee, Yih-Chih Kuo, Lo-Fan Cheng, Yi-Chieh Tsai, Jia-Zheng Huang, Hsin-Hsi Tsai, Jhih-Syuan Lin, Po-Ya Huang, Chen-Hung Ting, Chih-Chao Yang, Hsing-Jung Lai, Chi-Chao Chao, Li-Kai Tsai

**Affiliations:** 1 Department of Medical Imaging, National Taiwan University Hospital, Taipei 100225, Taiwan; bochinglee@gmail.com (B.-C.L.); eddy0407@ntuh.gov.tw (J.-Z.H.); 2 Department of Neurology, National Taiwan University Hospital and National Taiwan University College of Medicine, Taipei 100225, Taiwan; chichi8014@hotmail.com (Y.-C.K.); kevin850106@gmail.com (L.-F.C.); yct071525@gmail.com (Y.-C.T.); tsaihsinhsi@gmail.com (H.-H.T.); xup5@cycu.org.tw (J.-S.L.); huangpoya@hotmail.com (P.-Y.H.); jesse@ntuh.gov.tw (C.-C.Y.); i5492111@gmail.com (H.-J.L.); chichaochao@ntu.edu.tw (C.-C.C.); 3 Department of Neurology, National Taiwan University Hospital, Hsinchu Branch, Hsinchu City 300195, Taiwan; 4 Garage Brain Science, B201, Central Taiwan Innovation Campus, Ministry of Economic Affairs, Nantou City 540219, Taiwan; koichiting@gmail.com

**Keywords:** amyotrophic lateral sclerosis (ALS), animal model, enlarged perivascular space, magnetic resonance imaging (MRI), misfolded protein, superoxide dismutase type 1

## Abstract

Enlarged perivascular spaces (EPVS) are MRI markers of impaired glymphatic clearance and have been associated with neurodegenerative diseases. However, their clinical significance in amyotrophic lateral sclerosis (ALS) and underlying mechanisms remain poorly understood. This study investigated the prevalence, clinical relevance, and pathophysiological basis of EPVS in ALS. MRI data from 114 ALS patients and 119 matched controls were analyzed, with high-degree EPVS defined as more than 20 visible spaces. High-degree EPVS in the centrum semiovale (CSO) was more prevalent in ALS patients (49.1%) than in controls (15.1%, *p* < 0.001). Age, male sex, and ALS diagnosis were independent predictors, while disease severity and aggressiveness were not associated. ALS patients with high-degree CSO-EPVS were older at disease onset and MRI but showed similar clinical progression. In SOD1/G93A ALS mice, cerebral perivascular spaces were significantly enlarged at 5 months compared to wild-type and younger ALS mice. Cervical lymphatic ligation promoted misfolded SOD1 accumulation in motor neurons and cerebral vessels, further increasing perivascular space width without altering motor function. These findings suggest that about half of ALS patients exhibit high-degree CSO-EPVS, reflecting impaired protein clearance rather than disease aggressiveness.

## 1. Introduction

Amyotrophic lateral sclerosis (ALS) is a rare but devastating motor neuron disease without available curative treatment [1]. Patients with ALS eventually develop progressive degeneration of cortical and spinal motor neurons, leading to limb weakness, swallowing difficulty, slurred speech, and finally respiratory distress [2]. The mechanisms underlying ALS remain unknown, but insights from ALS genetics have shown that ALS involves dysregulation of numerous major cellular pathways, including dysfunction in protein quality control, RNA metabolism, and axonal trafficking [3]. The disturbance of proteostasis is a key pathological process in ALS in which misfolded protein disrupts cellular homeostasis through gain-of-toxicity, protein mislocalization, and interference with neuronal transport [4].

Perivascular spaces (PVS) are fluid-filled compartments that surround cerebral blood vessels, act as conduits for glymphatic fluid, and contribute to waste clearance in the brain [5]. PVS are considered pathologic when sufficiently enlarged to be visible on magnetic resonance imaging (MRI), referred to as enlarged perivascular space (EPVS), which may be a key indicator of glymphatic system dysfunction [6]. A notable example is Alzheimer’s disease, which is caused by the abnormal accumulation of misfolded beta-amyloid protein and is associated with EPVS, which correlates with cognitive impairment [7]. ALS, which also features aggregation of misfolded protein (such as TAR DNA-binding protein 43 [TDP-43] and superoxide dismutase type 1 [SOD1]), develops glymphatic dysfunction at the early stage of disease [8].

However, research focusing on the characteristics of EPVS in ALS is limited. Few studies have systematically characterized EPVS in ALS patients, and the pathological basis of these findings is poorly defined. Moreover, the relationship between EPVS, misfolded protein burden, and clinical outcomes in ALS has not been clarified. Therefore, the present study aimed to (1) determine the prevalence and clinical significance of EPVS in patients with ALS, and (2) investigate the potential mechanisms underlying EPVS using a transgenic ALS mouse model.

## 2. Results

### 2.1. Associations Between High-Degree CSO-EPVS and ALS

In 114 patients with ALS (age, 60.8 ± 13.3 years; woman, 47.5%), the onset age was 56.5 ± 23.0 years (range, 31–83 years) and 39 (34.2%) had bulbar onset ALS. The ALSFRS-R at evaluation was 34.0 ± 11.4 and the decline in ALSFRS-R per year was 10.5 ± 14.5. Seven patients (6.1%) were found to have the mutation of ALS-related genes, including SOD1 (*n* = 2), OPTN (*n* = 2), C9orf72 (*n* = 1), TARDBP (*n* = 1), and FUS (*n* = 1) genes. No patient had a history of stroke.

As compared to 119 controls (60.1 ± 12.3 years; female, 50.4%), patients with ALS showed comparable age, sex, and percentage of subjects having major underlying systemic diseases (Table 1). ALS patients had higher prevalence of high-degree CSO-EPVS than controls (49.1% vs. 15.1%, *p* < 0.001). No significant differences were found between patients with ALS and controls in prevalence of high-degree BG-EPVS, number of hippocampal EPVS, and prevalence of large EPVS. In the analysis of brain metrics, patients with ALS and controls had similar WMH, gray matter, and white matter volume. However, the cortical thickness was lower in patients with ALS than in controls for frontal (2.47 ± 0.16 vs. 2.53 ± 0.18 mm, *p* = 0.013), temporal (2.78 ± 0.20 vs. 2.83 ± 0.18 mm, *p* = 0.041), and occipital (2.00 ± 0.26 vs. 2.20 ± 0.21 mm, *p* < 0.001) lobes.

In univariable analysis, high-degree CSO-EPVS was significantly associated with older age, male sex, and ALS diagnosis (Table 2). In multivariable analysis, high-degree CSO-EPVS remained significantly associated with age (adjusted odds ratio [aOR] 1.589; 95% confidence interval [CI]: 1.176–2.147; *p* = 0.003), male sex (aOR 2.155; CI: 1.128–4.118; *p* = 0.020), and diagnosis of ALS (aOR 6.954; CI: 3.483–13.884; *p* < 0.001) after adjusting for age, sex, and underlying systemic diseases. Supplemental Figure S1 shows two examples of patients with ALS with high-degree CSO-EPVS.

### 2.2. Comparison Between Patients with ALS with and Without High-Degree CSO-EPVS

Characteristics of ALS patients with and without high-degree CSO-EPVS are compared (Table 3). ALS patients with high-degree CSO-EPVS were older at ALS onset (61.8 ± 10.1 vs. 51.3 ± 30.0, *p* = 0.014) and, at the time, they received MRI study (63.1 ± 10.3 vs. 57.6 ± 15.3, *p* = 0.028). However, no differences were found between ALS patients with and without high-degree CSO-EPVS in sex, onset pattern (bulbar onset or not), genetic mutation or not, disease severity (ALSFRS-R), or disease aggressiveness (annual ALSFRS-R decline). Kaplan–Meier analysis showed that the probabilities of being ventilator-free or surviving were also similar between the two groups (*p* = 0.363) (Supplemental Figure S2). After adjusting for age and sex, high-degree CSO-EPVS still did not show a higher risk of permanent ventilator support or mortality (Hazard ratio (HR) 0.712; 95% CI: 0.367–1.381). ALS patients with high-degree CSO-EPVS had similar WMH volume, gray, or white matter volume, and cortical thickness than those without high-degree CSO-EPVS (Table 3). However, higher volume of choroid plexus (1.5 ± 0.5 vs. 1.2 ± 0.5, *p* = 0.009) and the lateral ventricle (30.0 ± 20.0 vs. 21.8 ± 11.2, *p* = 0.028) were noted in patients with ALS than in those without high-degree CSO-EPVS. Nevertheless, after adjusting for age, sex, and ALSFRS-R, high-degree CSO-EPVS was not independently associated with higher volume of choroid plexus (OR, 1.000; 95% CI: 0.999–1.001) or the lateral ventricle (OR, 1.000; 95% CI: 1.000–1.000) in patients with ALS (Supplementary Table S1).

### 2.3. Increased Cerebral PVS Width and Misfolded Protein in ALS Mice

**Figure 1:**
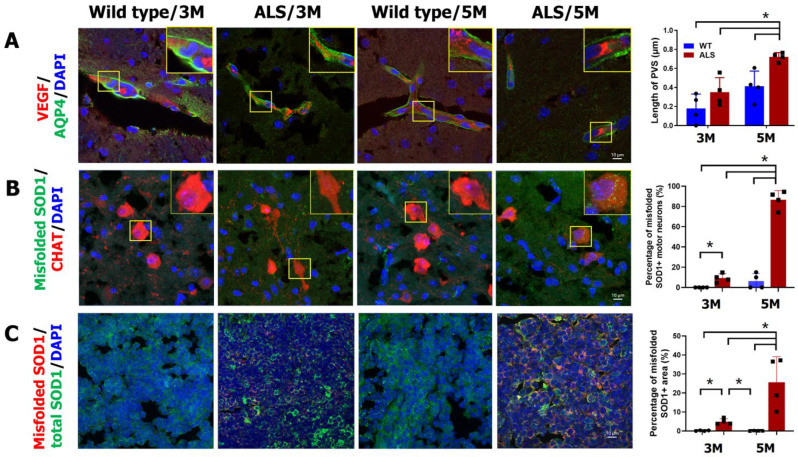
Perivascular Space (PVS) width and misfolded SOD1 distribution in ALS Mice. ALS/SOD1 and wild-type littermate mice were sacrificed at age 3 or 5 months for immunohistochemical staining. (**A**) Using anti-VEGF to label vessels (red, PVS inner margin) and anti-AQP4 to label astrocyte end-feet (green, PSV outer margin), the PVS is shown and the PVS width (vertical width between red and green margins) was analyzed and compared between groups, as shown. The nucleus is labeled with DAPI (blue). (**B**) The percentage of frontal motor neurons having intracellular misfolded SOD1 protein was analyzed and compared using anti-choline acetyltransferase (CHAT, red) to label motor neurons and anti-misfolded SOD1 (green) for misfolded protein with DAPI for nucleus (blue). (**C**) The deep cervical lymph nodes were stained for total SOD1 protein (green) and misfolded SOD1 protein (red) with DAPI for nucleus (blue). The percentage of misfolded SOD1-occupied areas among lymph node was analyzed and compared. Scale bar, 10 µm. *n* = 4 for each group. * *p* < 0.05; Kruskal–Wallis test with Mann–Whitney post hoc analysis.

PVS was analyzed in a mouse model of ALS with SOD1/G93A transgenes at age 3 months (early symptomatic stage) and 5 months (advanced stage). Wild-type littermates were used for comparison. Using anti-VEGF to label vessels (PVS inner margin) and anti-AQP4 for astrocyte end-feet (PSV outer margin), PVS width was measured using immunohistochemistry (Figure 1A). At age 3 months, only minimal PVS width was found in both ALS and wild-type mice (0.36 ± 0.14 and 0.18 ± 0.13 µm, respectively). The PVS became larger in ALS mice at age 5 months with PVS width 0.72 ± 0.04 µm, which was significantly higher than that in ALS mice at age 3 months (*p* = 0.029). However, the PVS remained small in wild-type mice at age 5 months with lower PVS width 0.41 ± 0.14 µm than that in ALS littermates (*p* = 0.029).

Abnormal protein aggregation in vessel walls is an important mechanism that drives EPVS formation [9,10]. Therefore, the expression of misfolded SOD1, a key component of protein aggregate in the SOD1/G93A model [11], was analyzed further in ALS and wild-type mice. At age 3 months, 9.30 ± 3.73% of frontal cortical motor neurons in ALS mice had intracellular misfolded SOD1 particles, which were higher than that in the wild-type littermates (0 ± 0%, *p* = 0.029) (Figure 1B). At age 5 months, the percentage of motor neurons in ALS mice having misfolded SOD1 particles (86.64 ± 7.81%) was significantly higher than that in both control littermates (6.25 ± 6.50%, *p* = 0.029) and ALS mice at age 3 months (*p* = 0.029). A previous study demonstrated that the glymphatic system contributes to the clearance of beta-amyloid from the human brain in part via the cervical lymphatic system [12]. Further analysis of the distribution of total and misfolded SOD1 in the cervical lymph nodes of ALS and wild-type mice showed that the amount of total SOD1 protein in the cervical lymph node was similar in ALS and wild-type mice regardless of age. Misfolded SOD1 protein appeared in cervical lymph nodes in ALS mice with an increase in percentages of misfolded SOD1-occupied area among lymph nodes from ages 3 to 5 months (4.91 ± 1.32% vs. 25.65 ± 11.64%, *p* = 0.029) (Figure 1C). In contrast, nearly no misfolded SOD1 signal was found in cervical lymph nodes of wild-type mice. Taken together, the changes in PVS width seem to parallel the changes in the amount of misfolded SOD1 in motor neurons and cervical lymph nodes.

### 2.4. Pathology and Behaviors After Ligation of Cervical Lymphatic Vessels in ALS Mice

**Figure 2:**
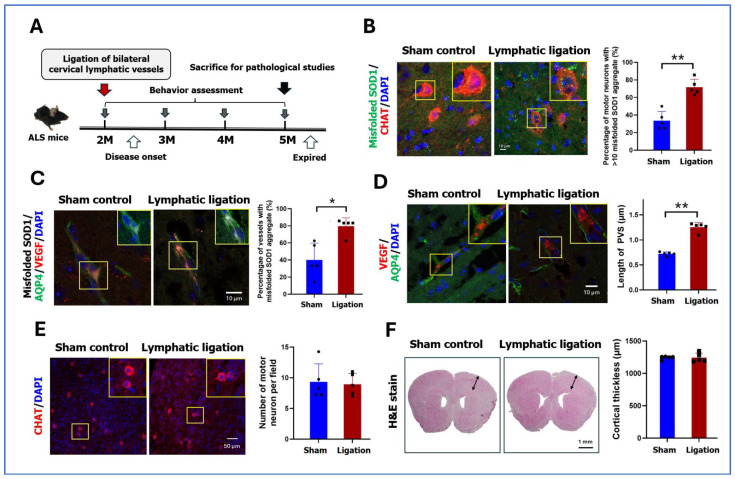
Pathology and behaviors after ligation of cervical lymphatic vessels in ALS mice. (**A**) ALS mice receiving bilateral cervical lymphatic vessel ligation or not at age 2 months were evaluated for functional behaviors monthly, including rotarod maintenance test and Y-maz spontaneous alternation test and sacrificed at age 5 months for brain pathology analysis. (**B**) Percentage of frontal motor neurons having >10 intracellular misfolded SOD1 protein aggregates were analyzed and compared using anti-CHAT (red) and anti-misfolded SOD1 (green) with DAPI (blue). Scale bar, 10 µm. (**C**) The percentage of VEGF labeled-cerebral vessel (red) having condense misfolded SOD1 protein aggregate (white) was analyzed and compared with anti-AQP4 labeling for astrocyte end-feet (green) and DAPI for nucleus (blue). Scale bar, 10 µm. (**D**) The width of PVS (vertical width between red [VEGF-labeling] and green [AQP4-labeling] margins) was analyzed and compared between two groups. Nucleus was labeled with DAPI (blue). Scale bar, 10 µm. (**E**) The density of CHAT-labeled motor neurons (red) in the motor cortex was analyzed. Nucleus was labeled with DAPI (blue). Scale bar, 50 µm. (**F**) The frontal cortical thickness (double arrow; width between brain surface and cortico-striatal border) was measured using hematoxylin and eosin staining. Scale bar, 1 mm; *n* = 5 for each group in above pathological analysis. * *p* < 0.05, ** *p* < 0.01; Mann–Whitney U test.

Bilateral cervical lymphatic vessels were ligated in ALS mice to diminish the misfolded SOD1 outflow to study the influence of misfolded protein accumulation on EPVS (Figure 2A). ALS mice receiving bilateral cervical lymphatic ligation or not at age 2 months were sacrificed at age 5 months for brain pathology analysis. ALS mice receiving cervical lymphatic vessel ligation showed higher amounts of misfolded SOD1 aggregation than those without ligation, not only in cortical motor neurons (100.00 ± 0% vs. 93.68 ± 3.93% of neurons having any misfolded SOD1 particle, *p* = 0.048) (71.79 ± 7.95% vs. 33.65 ± 9.38% of neurons having >10 misfolded SOD1 particles, *p* = 0.008) (Figure 2B) but also in cerebral vessels (79.6 ± 8.6% vs. 40.1 ± 17.6% of vessels having condense misfolded SOD1, *p* = 0.016) (Figure 2C). In addition, ALS mice with lymphatic vessel ligation had significantly larger PVS width than those without (1.26 ± 0.08 vs. 0.72 ± 0.35 µm, *p* = 0.008) (Figure 2D). Further analysis of the consequences of bilateral cervical lymphatic vessel ligation on pathological and functional outcomes in ALS mice showed that at three months after lymphatic ligation, the density of cortical motor neurons was similar between ALS mice with and without ligation (Figure 2E). The frontal cortical thickness was also comparable between the two groups (Figure 2F). Moreover, ALS mice receiving lymphatic vessel ligation showed similar behaviors as those without ligation using fixed and accelerated modes of rotarod maintenance test for assessment of motor function (Supplemental Figure S3A) as well as Y-maz spontaneous alternation test for the analysis of memory function (Supplemental Figure S3B).

## 3. Discussion

The prevalence, significance, and mechanisms of cerebral EPVS in ALS are still unclear. In the present study, nearly half (49.1%) of ALS patients had high-degree CSO-EPVS visualized on brain MRI, showing that prevalence is much higher in the patients with ALS than that in the control subjects (15.1%). After adjusting for age, sex, and underlying diseases, a diagnosis of ALS remains a significant determinant for developing high-degree CSO-EPVS. The clinical features, including disease severity and aggressiveness, are similar between ALS patients with and without high-degree CSO-EPVS, except the former are relatively older at disease onset and at study enrollment. In addition, ALS mice at the advanced disease stage also had EPVS; the appearance of EPVS is parallel to the amount of misfolded SOD1. Bilateral cervical lymphatic vessel ligation in ALS mice results in the accumulation of misfolded SOD1 in motor neurons and cerebral vessels, which are associated with the increased severity of EPVS, but do not influence pathological or functional outcomes. Taken together, high-degree CSO-EPVS is not uncommon in ALS patients, which is not associated with disease aggressiveness and likely caused by the accumulation of cerebral misfolded protein.

The common imaging features for ALS, as visualized by MRI, include frontal-predominant brain atrophy, T2 hypointensity in the motor cortex, and T2 hyperintensity and reduction in fractional anisotropy in the corticospinal tracts [13]. The present study is the first to demonstrate that half of ALS patients have high-degree CSO-EPVS that can be detected using T2-weighted imaging of conventional MRI. Since EPVS is thought to be an indicator of glymphatic dysfunction, our result is in line with the observation of glymphatic dysfunction in ALS patients [8]. The EPVS in ALS is mainly located at CSO rather than BG or the hippocampus, possibly because the frontal lobe is the primary location of misfolded protein deposition in ALS [14]. Although age, male sex, and various systemic diseases are risk factors for EPVS [15,16,17], ALS diagnosis remains an independent determinant after adjusting for these factors. Since no specific clinical feature of ALS is associated with high-degree CSO-EPVS, this potential MRI biomarker (high-degree CSO-EPVS) cannot be applied to predict disease pattern, severity, or aggressiveness.

High-degree CSO-EPVS can also be detected in patients with other neurodegenerative diseases, such as Alzheimer’s disease (39.1–44.7%) and Parkinson’s disease (42.4–76%) [7,18,19,20], and cerebrovascular diseases, such as cerebral amyloid angiopathy (43.8–44.8%) [21,22]. The common features of the above diseases, including ALS, involve the existence of misfolded protein aggregation in specific brain regions. PVS serve as conduits for interstitial fluid and waste clearance via the glymphatic system [5]. One of the important mechanisms behind the development of EPVS is excessive protein aggregation in vessel walls [9,10], which could contribute to perivascular fluid stasis and enlargement visible on MRI. Our animal findings support this mechanism, as the changes in width of PVS were parallel to changes in the amount of misfolded SOD1 in the motor neurons and cervical lymph nodes of ALS mice. In addition, ligation of bilateral cervical lymphatic vessels in ALS mice enhanced accumulation of misfolded SOD1 in cerebral vessels, which is associated with increased severity of EPVS, suggesting that protein aggregates may accumulate within perivascular spaces when clearance is impaired. Collectively, the EPVS in ALS is likely caused by accumulation of misfolded protein in the cerebral vessels. This mechanism resembles cerebral amyloid angiopathy in Alzheimer’s disease, where β-amyloid deposition in vessel walls promotes EPVS formation [22]; however, in ALS, the protein species are predominantly SOD1 and TDP-43. Nevertheless, half of patients with ALS did not have high-degree CSO-EPVS. The potential disease mechanisms of ALS include not only dysfunction in protein homeostasis leading to misfolded protein aggregation, but also impairment in RNA metabolism and axonal trafficking, neuroinflammation, mitochondrial dysfunction, oxidative stress, and excitotoxicity, etc. [23]. Each patient may have an individual–specific combination of different mechanisms underlying ALS development. It remains possible that ALS patients in whom protein homeostasis dysfunction plays a major role in the disease mechanism may be more likely to have high-degree CSO-EPVS.

ALS patients with high-degree CSO-EPVS did not display more rapid progression or worse survival than those without high-degree CSO-EPVS. The high-degree CSO-EPVS was also not associated with gray or white matter atrophy or reduced cortical thickness. ALS mice with lymphatic vessel ligation had a higher degree of EPVS but did not show lower density of motor neurons in the frontal cortex, more cortical atrophy, worse motor behaviors, or poorer memory function than those without ligation surgery. In contrast, the appearance of high-degree CSO-EPVS correlated well with cognitive impairment in Alzheimer’s disease [7]. High-degree BG-EPVS in Parkinson’s disease was also associated with cognitive dysfunction in language and visual memory [19]. In ALS, progression is primarily driven by motor neuron loss rather than cortical-subcortical network disruption, which may explain why EPVS does not predict aggressiveness or prognosis. Thus, in ALS, CSO-EPVS may represent an epiphenomenon of protein accumulation and impaired clearance rather than a driver of functional decline.

The choroid plexus, as a part of the glymphatic system, contributes to clear harmful waste from the brain [24]. The choroid plexus volume was greater in patients with Alzheimer’s disease than healthy controls and was correlated with the global burden of beta-amyloid deposition [25,26]. In addition, the ventricular volume correlated positively with the choroid plexus volume in patients with Alzheimer’s disease [27]. Previous research indicates that beta-amyloid accumulation in the choroid plexus affects its structure and function, with enlarged choroid plexus and blood-CSF barrier disruption, and subsequent further reduction in CSF beta-amyloid clearance [28]. Although we also found that the appearance of high-degree CSO-EPVS was associated with an increase in the volume of choroidal plexus and lateral ventricle in patients with ALS, high-degree CSO-EPVS was not an independent determinant of higher volume of choroid plexus or lateral ventricle after adjusting for age, sex, and ALS severity in multivariable analysis.

This study has several limitations. Although the data were collected prospectively from the ALS cohort, this study was designed retrospectively. Therefore, aggressiveness of treatment and measurement of outcomes throughout the database would probably be less consistent than those achieved with a prospective cohort study. In addition, the number of patients was relatively small, all subjects came from a single center, and all patients with ALS were Asian, which potentially limits generalizability to other populations and ethnicities. The research was designed as a cross-sectional MRI study without imaging follow-up, so serial changes in CSO-EPVS and their clinical correlations over time remain unclear. Taken together, future large international studies with longitudinal follow-up of MRI are needed to thoroughly analyze the changes in high-degree CSO-EPVS in ALS patients. In addition, more than 90% of sporadic patients with ALS are shown to have TDP-43 aggregation in motor neurons [29]; however, we used ALS/SOD1 mice and analyzed the misfolded SOD1 protein for mechanisms underlying EPVS in ALS. Finally, the results of this study did not validate the results in human brain pathology, suggesting that future research using human brain banks or plasma/CSF biomarkers would be useful.

## 4. Materials and Methods

### 4.1. Study Design and Sample Selection

For this retrospective cohort study, the data of consecutive patients with ALS diagnosed and treated in the Department of Neurology, National Taiwan University Hospital, Taipei, Taiwan, from October 2017 to May 2024, and those who underwent brain MRI study, were included in the ALS cohort. Probable ALS was diagnosed based on the revised Awaji ALS diagnostic criteria [30]. Patients received genetic testing to evaluate at least four common ALS family genes, including SOD1, C9orf72, TARDBP, and FUS. All patients underwent clinical history taking, neurological examination, and questionnaires, including the revised ALS functional rating scale (ALSFRS-R), and MRI. The decline in ALSFRS-R per year was used to evaluate the rate of disease progression. The rate of decline was determined using the established formula [31]:

(48 − ALSFRS-R score)/(disease duration from initial symptom onset to evaluation in years)

The control group consisted of 119 subjects matched for age and sex, who did not have any neurological disease. These controls had undergone an MRI study for diagnostic purposes because of various symptoms, including headache (*n* = 24), tremor (*n* = 22), equivocal stroke-like symptoms (*n* = 17), dizziness (*n* = 15), nonspecific memory complaints (*n* = 9), nonspecific unsteadiness (*n* = 6), facial pain (*n* = 4), tinnitus (*n* = 1), concussion (*n* = 1), or miscellaneous (*n* = 20).

The human study protocol was approved by the Research Ethics Committee of National Taiwan University Hospital (#201810041RINC, #202107090RINA, date of approval: 1 July 2022), with informed consent waived. Animal experiments were approved by the Institutional Animal Care and Use Committee of the National Taiwan University College of Medicine Laboratory Animal Center (#20210194).

### 4.2. MRI Acquisition and Analysis

Brain MRIs were obtained using a 1.5T or 3T MRI scanner (MAGNETOM Aera, MAGNETOM Verio, TIM, or Biograph mMR; all from Siemens Medical Solutions, Malvern, PA, USA). The imaging protocols included a T1-weighted magnetization-prepared rapid gradient-echo imaging (MPRAGE, flip angle 9, repetition time/echo time = 1460/2.39 ms, field of view = 25.6 cm and slice thickness = 1 mm), T2-weighted imaging (repetition time/echo time = 3530/83 ms, field of view = 23 cm, and slice thickness = 5 mm), fluid-attenuated inversion recovery imaging (FLAIR; repetition time/echo time = 10,000/89 ms, field of view = 23 cm and slice thickness = 5 mm), diffusion-weighted imaging, and apparent diffusion coefficient maps. MRI markers related to cerebral small vessel disease were evaluated based on the Standards for Reporting Vascular Changes on Neuroimaging criteria (STRIVE2) [32]. MRI-visible PVSs, representing EPVS, were evaluated on T2-weighted imaging and defined as sharply delineated structures measuring < 3 mm, following the course of perforating or medullary vessels by the neuroradiologist, as previously described [21]. High-degree EPVS was defined as >20 visible PVSs in the centrum semiovale (CSO) or in the basal ganglia (BG) on the side of the brain with more severe involvement [33]. In addition, the presence of large PVS in the BG, defined as round or tubular structures with a short axis greater than 3 mm, lacking a rim or high signal intensity was evaluated using axial FLAIR MRI and without hemosiderin evidence on axial SWI, as previously described [34]. White matter hyperintensity (WMH) volume was analyzed based on FLAIR imaging. All MRI scans were also processed using FreeSurfer software v6.0.0 (http://surfer.nmr.mgh.harvard.edu/, accessed on 1 June 2019) to determine WMH volume, the gray, and white matter volume, cerebral cortical thickness of total and individual lobes, choroid plexus volume, and volume of ventricles using a T1-weighted MPRAGE sequence.

### 4.3. Animal Model

A mouse model of ALS carrying the mutant human SOD1/G93A transgenes (B6SJL-Tg(SOD1∗G93A)1Gur/J) was maintained under a 12/12 h light–dark cycle, following standard procedures at National Taiwan University College of Medicine Laboratory Animal Center. These ALS mice showed disease onset at 2.5–3 months and expired at 5–6 months of age, as demonstrated in our previous study [35]. To investigate PVS and misfolded protein, ALS and wild-type littermate mice at ages of 3 and 5 months were sacrificed and subjected to a pathological analysis of brain and deep cervical lymph nodes.

To investigate the mechanism of EPVS in ALS mice, we tried to inhibit the misfolded SOD1 outflow to study the influence of misfolded protein accumulation on EPVS. The bilateral lymphatics afferent to the deep cervical lymph nodes of ALS mice were ligated to analyze the effects of lymphatic obstruction as described previously [36]. Briefly, mice at age 2 months were anesthetized with intraperitoneal injection of Zoletil. After a midline incision 5 mm superior to the clavicle was made, collecting lymphatic vessels anterior to the deep cervical lymph nodes were ligated using an 8-0 nylon suture. The mice were then sutured and were subjected to serial behavioral tests per month and sacrificed at age 5 months for pathology studies. Sham-controlled ALS littermates receiving the same midline excision but without lymphatic vessel ligation were used for comparison.

### 4.4. Pathological Studies of ALS Mice

Mice were anesthetized with a lethal dose of Zoletil and fixed by transcardial perfusion with saline, followed by 4% paraformaldehyde. The brains and cervical lymph nodes were submerged in 15% then 30% sucrose, and frozen at −80 °C. A series of 10 µm thick coronal sections were cryo-cut at the anterior brain including motor cortex and striatum. The samples were blocked with 5% normal goat/rabbit serum (Sigma-Aldrich, St. Louis, MO, USA) for one hour at room temperature, and then incubated overnight at 4 °C with the following primary antibodies: anti-vascular endothelial growth factor with fluorescence conjugated (VEGF; 1:100; Novus Biologicals, #NB100-664AF647, Minneapolis, MN, USA), anti-VEGF (1:100; Invitrogen, #MA1-16629, Waltham, MA, USA), anti-aquaporin 4 (AQP4; 1:1000; Novus Biologicals, #NBP1-87679), anti-SOD1 (1:100; Cell Signaling, #37385, Danvers, MA, USA), anti-misfolded SOD1 (1:100, MEDIMABS, #MM-0070-P, Montreal, QC, Canada), and anti-choline acetyltransferase (CHAT; 1:100, Merck, #ABP-144P, Darmstadt, Germany). Samples were then washed and incubated for two hours at room temperature with the corresponding secondary antibody conjugated with Alexa Fluor 546 or 488 (1:200; Thermo Fisher Scientific, Waltham, MA, USA) for 30 min. Nuclei were stained with 4′, 6-diamidino-2-phenylindole (DAPI). Fluorescent labeling was examined with a laser-scanning confocal microscope (LSM880 confocal microscope; Carl Zeiss, Oberkochen, Germany) with either a 20× or 40× objective lens. Some brain sections were sent to Laboratory Animal Center of National Taiwan University College of Medicine, Taipei, Taiwan, to process hematoxylin and eosin staining for measurement of cortical thickness, which was detected using Nikon ECLIPSE Ti2-U (Tokyo, Japan) and Olympus SZ61 (Tokyo, Japan) optical microscopies.

Quantitative analyses included the vertical length between VEGF (PVS inner margin) and AQP4 (PVS outer margin) signals representing PVS width in cerebral cortex (*n* = 25 per mouse), percentage of CHAT-positive motor neurons having any or more than 10 misfolded SOD1 particles (*n* > 15 per mouse), number of CHAT-positive motor neurons per random field in motor cortex (*n* = 5 per mouse), percentage of misfolded SOD1-positive area among lymph node area (*n* = 5 per mouse), percentage of vessel having condense misfolded SOD1 signals (*n* > 7 per mouse), and vertical length between brain surface and cortical-striatal junction representing cortical thickness (*n* = 6 per mouse).

### 4.5. Behavioral Studies of ALS Mice

Motor functional performances using the rotarod maintenance test were analyzed monthly in ALS mice with and without cervical lymphatic vessel ligation from the age of 2 to 5 months, as previously described [37]. Briefly, the mice were trained three times by placing them in a neutral position on a stationary 3 cm diameter cylinder of the rotarod apparatus (RT-01, SINGA, Diagnostic & Research Instruments Co., Taoyuan, Taiwan) with a constant rotation speed of 5 rpm for 30 s. Mice were given 30 min breaks between the training and test phases. During the test phase, each mouse was subjected to two modes of analysis: fixed and accelerated. The constant rotating speed was set at 20 rpm in the fixed mode, while the accelerated speed was set from 5 rpm to 40 rpm in 5 min in the accelerated mode. The time taken to fall was recorded when each mouse fell off the rod. Three tests were performed, with averages for each condition.

In addition, Y-maze spontaneous alternation test was applied to study the memory function of ALS mice [38]. Prior to initiating the behavioral tests, we allowed the animals to acclimatize in the testing room for a minimum of 2 min. During the test phase, mice were placed in the Y-maze for 10 min. An entry and an exit of an arm were defined as when both the head and the tail base crossed the boundary of the center zone. Each arm choice was classified correct, incorrect, or neutral. If the last and second last-visited arms were different and the current choice was different from those two arms, then it was considered a correct choice. If the mice chose the other arms, the choice was considered incorrect. If the last and second last-visited arms were the same, then the current arm choice was considered neutral. The spontaneous alternation rate was calculated as the number of correct choices divided by the total number of correct and incorrect choices.

### 4.6. Statistical Analysis

Categorical variables are presented as percentages, and continuous variables are presented as mean ± standard deviation. In human studies, baseline demographic and clinical characteristics, and neuroimaging variables were compared between ALS patients and control subjects and between ALS patients with and without high-degree CSO-EPVS using the independent sample *t*-test or chi-square test as appropriate. A multivariable logistic regression model was built to examine the independent associations between high-degree CSO-EPVS and relevant demographic and clinical characteristics (age, sex, and the presence/absence of hypertension, diabetes mellitus, chronic kidney disease, and coronary artery disease) and diagnosis of ALS. Another multivariable logistic regression model was built to examine the independent associations in ALS patients between high-degree CSO-EPVS and relevant outcomes (ALSFRS-R annual decline, gray and white matter volume, WMH volume, cortical thickness, and volume of choroid plexus and lateral ventricle) after adjusting for age, sex, and ALSFRS-R. The Kaplan–Meier analysis was conducted to plot the permanent ventilator-free survival probability, and the log-rank test was applied to test the difference between patients with and without high-degree CSO-EPVS. In addition, Cox regression analyses were used to calculate the adjusted (age, sex) hazard ratios (HRs) for the occurrence of requiring permanent ventilator or death. In animal studies, the means between four groups were compared using the Kruskal–Wallis H test followed by the Mann–Whitney U post hoc analysis and between two groups using Mann–Whitney U test. Two-tailed *p*-values < 0.05 were established as statistical significance. All statistical analysis was conducted using SPSS version 17 (SPSS, Inc., Chicago, IL, USA) and PRISM version 9 (GraphPad Software Inc., Irvine, CA, USA).

## 5. Conclusions

Overall, the results obtained in this study suggest that high-degree CSO-EPVS is an important imaging feature in ALS, which appears in about half of patients. The high-degree CSO-EPVS is likely caused by accumulation of misfolded protein in the cerebral vessels. Therefore, the existence of high-degree CSO-EPVS might potentially imply the high misfolded protein burden in the brain, which should be validated in further human pathological studies. In addition, the existence of high-degree CSO-EPVS is not associated with unfavorable outcomes in ALS, unlike Alzheimer’s disease or Parkinson’s disease, in which high-degree EPVS correlates with poor prognosis.

Associations between CSO-EPVS, the glymphatic system, misfolded protein, and clinical outcomes in ALS required future investigation in large, prospective, multicenter cohort studies. Multimodal imaging may further establish CSO-EPVS as a noninvasive biomarker to explore disease mechanisms and monitor therapeutic response.

## Figures and Tables

**Table 1:** Comparison of demographic and neuroimaging characteristics between patients with ALS and healthy controls.

	ALS (*n* = 114)	Controls (*n* = 119)	*p* Value
Clinical characteristics			
Age, year	60.8 ± 13.3	60.1 ± 12.3	0.674
Sex, male	61 (53.5%)	59 (49.6%)	0.549
Hypertension	40 (35.1%)	34 (28.6%)	0.285
Diabetes mellitus	9 (7.9%)	15 (12.6%)	0.237
Chronic kidney disease	1 (0.8%)	2 (1.7%)	0.587
Coronary artery disease	6 (5.3%)	8 (6.7%)	0.639
Perivascular space			
BG-EPVS > 20, *n* (%)	18 (15.8%)	12 (10.1%)	0.194
CSO-EPVS > 20, *n* (%)	56 (49.1%)	18 (15.1%)	<0.001
Hippocampal EPVS, *n*	3.8 ± 2.6	3.8 ± 1.7	0.860
Large EPVS, *n* (%)	13 (11.4%)	8 (6.7%)	0.212
Whole brain metrics			
White matter hyperintensity (mL)	5.4 ± 8.7	3.5 ± 5.7	0.055
Gray matter volume (mL)	586.3 ± 51.5	584.8 ± 54.2	0.845
White matter volume (mL)	470.8 ± 57.2	467.7 ± 66.0	0.735
Cortical thickness			
Total (mm)	2.42 ± 0.14	2.46 ± 0.14	0.298
Frontal lobe (mm)	2.47 ± 0.16	2.53 ± 0.18	0.013
Temporal lobe (mm)	2.78 ± 0.20	2.83 ± 0.18	0.041
Parietal lobe (mm)	2.21 ± 0.16	2.24 ± 0.19	0.239
Occipital lobe (mm)	2.00 ± 0.26	2.20 ± 0.21	<0.001

Abbreviations: ALS, amyotrophic lateral sclerosis; BG, basal ganglion; CSO, centrum semiovale; EPVS, enlarged perivascular space.

**Table 2:** Logistic regression analysis of associations between high-degree CSO-EPVS and demographic and neuroimaging characteristics.

	Univariable Logistic Regression			Multivariable Logistic Regression		
	OR	95% CI	*p* Value	OR	95% CI	*p* Value
Age, per 10 years	1.463	1.142–1.874	0.003	1.589	1.176–2.147	0.003
Male sex	2.051	1.163–3.615	0.013	2.155	1.128–4.118	0.020
ALS	5.418	2.910–10.086	<0.001	6.954	3.483–13.884	<0.001
Hypertension	1.145	0.636–2.061	0.651	0.48	0.221–1.039	0.062
Diabetes mellitus	1.083	0.442–2.658	0.861	1.261	0.422–3.764	0.678
Chronic kidney disease	4.389	0.392–49.188	0.230	5.505	0.470–64.532	0.174
Coronary artery disease	2.269	0.766–6.723	0.139	2.978	0.811–10.931	0.100

Abbreviations: ALS, amyotrophic lateral sclerosis; CI, confidence interval; CSO, centrum semiovale; EPVS, enlarged perivascular space; OR, odds ratio.

**Table 3:** Comparison of high-degree CSO-EPVS (with/without) in patients with ALS.

	With High-Degree CSO-EPVS (*n* = 56)	Without High-Degree CSO-EPVS (*n* = 58)	*p* Value
Clinical characteristics			
Age of ALS onset, year	61.8 ± 10.1	51.3 ± 30.0	0.014
Age of MRI study, year	63.1 ± 10.3	57.6 ± 15.3	0.028
Sex, male	35 (62.5%)	26 (44.8%)	0.059
Bulbar onset	23 (41.1%)	16 (27.6%)	0.129
Mutation of ALS-related genes	3 (5.4%)	4 (6.9%)	1.000
ALSFRS-R	35.8 ± 10.1	32.2 ± 12.4	0.088
ALSFRS-R decline (1/year)	10.9 ± 18.8	10.2 ± 9.0	0.798
Neuroimaging features			
White matter hyperintensity (mL)	5.8 ± 9.2	5.0 ± 8.3	0.606
Gray matter volume (mL)	580.9 ± 49.6	591.5 ± 53.4	0.358
White matter volume (mL)	468.4 ± 62.8	473.1 ± 51.8	0.714
Cortical thickness (mm)			
Total	2.40 ± 0.16	2.44 ± 0.11	0.123
Frontal lobe	2.44 ± 0.19	2.50 ± 0.13	0.068
Temporal lobe	2.75 ± 0.25	2.80 ± 0.14	0.227
Parietal lobe	2.18± 0.15	2.24 ± 0.15	0.078
Occipital lobe	1.96 ± 0.27	2.05 ± 0.24	0.146
Choroid plexus volume (mL)	1.5 ± 0.5	1.2 ± 0.5	0.009
Lateral ventricle volume (mL)	30.0 ± 20.0	21.8 ± 11.2	0.028

Abbreviations: ALS, amyotrophic lateral sclerosis; ALSFRS-R, revised ALS functional rating scale; CSO, centrum semiovale; EPVS, enlarged perivascular space; MRI, magnetic resonance imaging.

## Data Availability

Anonymized data not published within this article will be made available by request from any qualified investigator.

## Supplementary Materials

The following supporting information can be downloaded at: https://www.mdpi.com/article/10.3390/ijms26199474/s1.

## Abbreviations

ALS, amyotrophic lateral sclerosis; ALSFRS-R, revised amyotrophic lateral sclerosis functional rating scale; AQP4, aquaporin 4; BG, basal ganglion; CHAT, choline acetyltransferase; CI, confidence interval; CSO, centrum semiovale; CSF, cerebrospinal fluid; DAPI, 4′,6-diamidino-2-phenylindole; DM, diabetes mellitus; EPVS, enlarged perivascular space; EB, Evans Blue; MRI, magnetic resonance imaging; OD, odds ratio; PVS, perivascular space; SOD-1, superoxide dismutase type 1; TDP-43, TAR DNA-binding protein 43; VEGF, vascular endothelial growth factor; WMH, white matter hyperintensity.
