# Supplementary material for: The Significance and Mechanism of Cerebral Enlarged Perivascular Space in Amyotrophic Lateral Sclerosis
Source: Int J Mol Sci. 2025 Sep 27;26(19):9474. doi: 10.3390/ijms26199474 (PMC12524775; doi:10.3390/ijms26199474)
Supplement: Supplementary file 1 [file ijms-26-09474-s001.zip › ijms-3884888-supplementary.pdf]

Supplementary Materials:

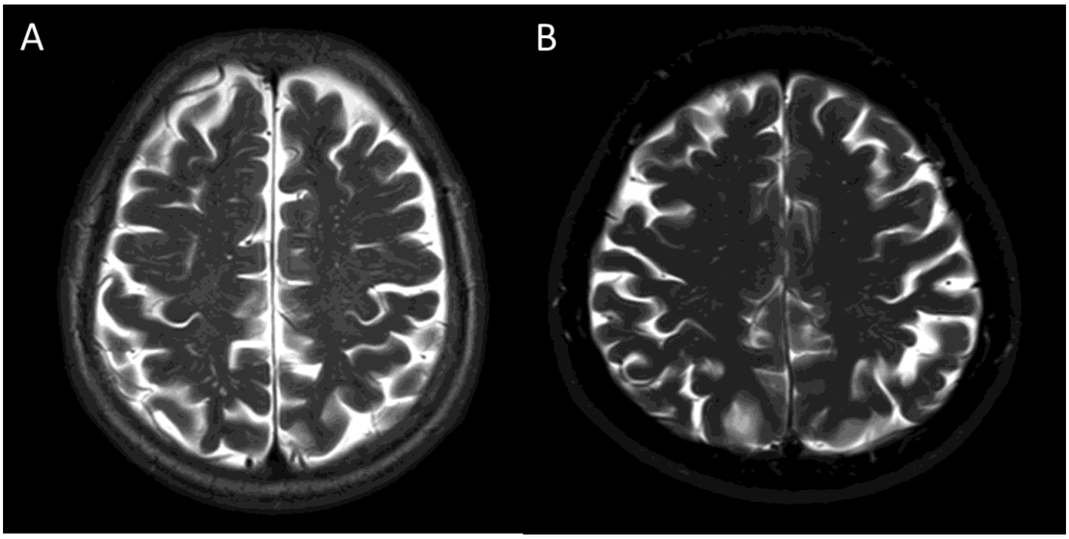

Supplemental Figure S1 The presence of high-degree CSO-EPVS in patients with ALS: a middle-aged female (A) and a middle-aged male (B), respectively.

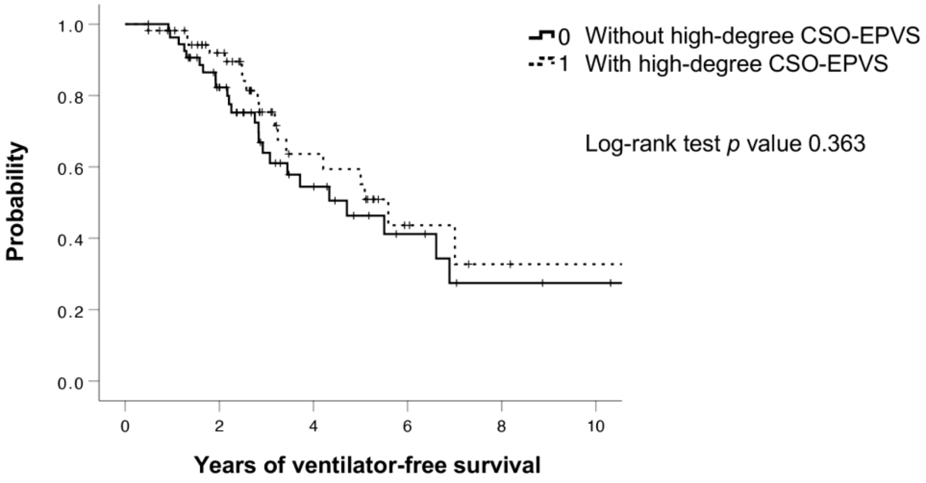

Supplemental Figure S2 Kaplan-Maier survival curve analyzing ventilator-free survival for patients with ALS stratified by presence of high-degree CSO-EPVS or not.

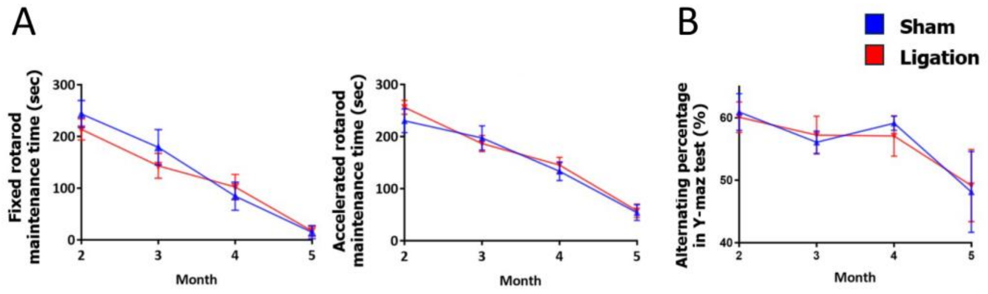

Supplemental Figure S3 (A) The results of rotarod maintenance test in ALS mice with (n=14) and without (n=12) receiving lymphatic ligation from the age of 2 to 5 months using fixed or accelerated mode were shown. (B) The results of Y-maz spontaneous alternation test were shown

Supplemental Table S1. Logistic Regression Analysis Investigating Associations between High-degree CSO-EPVS and Clinical and Neuroimaging Outcomes in Patients with ALS.
Multivariable logistic regression analysis after adjusting for age, sex, and ALSFRS-R.

	Univariable logistic regression			Multivariable logistic regression		
	OR	95% CI	p Value	OR	95% CI	p Value
ALSFRS-R decline	1.004	(0.978-1.031)	0.765	1.011	(0.980-1.043)	0.495
Grey matter volume	1.000	(1.000-1.000)	0.354	1.000	(1.000-1.000)	0.182
White matter volume	1.000	(1.000-1.000)	0.710	1.000	(1.000-1.000)	0.116
WMH volume	1.011	(0.969-1.056)	0.605	0.995	(0.947-1.046)	0.854
Total cortical thickness	0.070	(0.002-2.216)	0.131	0.896	(0.017-47.934)	0.957
Frontal cortical thickness	0.066	(0.003-1.292)	0.073	0.453	(0.013-15.912)	0.663
Choroid plexus volume	1.001	(1.000-1.002)	0.013	1.000	(0.999-1.001)	0.891
Lateral ventricle volume	1.000	(1.000-1.000)	0.039	1.000	(1.000-1.000)	0.776
